# Diethyl 2-oxo-3-(2-oxo-2,3-dihydro-1*H*-indol-3-yl­idene)butane­dioate

**DOI:** 10.1107/S160053680905140X

**Published:** 2009-12-04

**Authors:** I. K. LeninTamikovan, A. SubbiahPandi, M. Damodiran, K. Karthikeyan, P. T. Perumal

**Affiliations:** a Tamilnadu Science and Technology Centre, Chennai 600 025, India; b Department of Physics, Presidency College (Autonomous), Chennai 600 005, India; c Organic Chemistry Division, Central Leather Research Institute, Chennai 600 020, India

## Abstract

The title compound, C_16_H_15_NO_6_, crystallizes with two symmetry–independent mol­ecules in the asymmetric unit. The crystal structure is stabilized by inter­molecular C—H⋯O and N—H⋯O hydrogen bonds, and intra­molecular C—H⋯O hydrogen bonds. In addition, the crystal structure exhibits two inter­molecular C—H⋯π inter­actions.

## Related literature

For the use of indole derivatives as bioactive drugs, see: Stevenson *et al.* (2000 ▶). They exhibit anti-allergic, central nervous system depressant and muscle-relaxant properties, see: Harris & Uhle (1960 ▶); Ho *et al.* (1986 ▶). Indoles also exhibit high aldose reductase inhibitory activity, see: Rajeswaran *et al.* (1999 ▶). For bond-length data, see: Allen *et al.* (1987 ▶). For hydrogen-bond motifs, see: Bernstein *et al.* (1995 ▶).
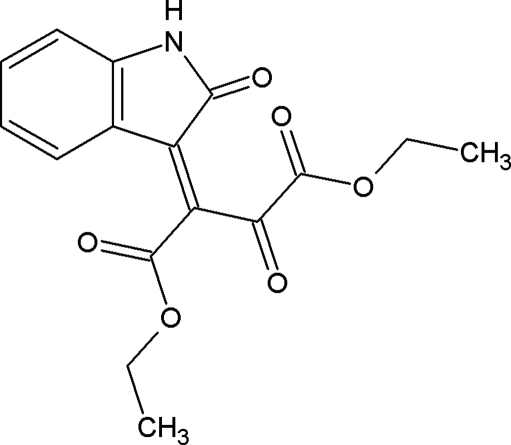


## Experimental

### 

#### Crystal data

C_16_H_15_NO_6_
*M* *_r_* = 317.29
Triclinic, 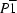

*a* = 8.8277 (2) Å
*b* = 13.5365 (4) Å
*c* = 13.6300 (3) Å
α = 96.516 (3)°
β = 102.218 (2)°
γ = 100.668 (1)°
*V* = 1544.44 (7) Å^3^
*Z* = 4
Mo *K*α radiation
μ = 0.11 mm^−1^
*T* = 293 K
0.24 × 0.22 × 0.16 mm

#### Data collection

Bruker APEXII CCD diffractometer
Absorption correction: multi-scan (*SADABS*; Sheldrick, 2001 ▶) *T*_min_ = 0.975, *T*_max_ = 0.983
37712 measured reflections
8406 independent reflections
5743 reflections with *I* > 2σ(*I*)
*R*_int_ = 0.028

#### Refinement

*R*[*F* ^2^ > 2σ(*F* ^2^)] = 0.056
*wR*(*F* ^2^) = 0.190
*S* = 1.02
8406 reflections
420 parameters
H-atom parameters constrained
Δρ_max_ = 0.60 e Å^−3^
Δρ_min_ = −0.35 e Å^−3^

### 

Data collection: *APEX2* (Bruker, 2004 ▶); cell refinement: *APEX2* and *SAINT* (Bruker, 2004 ▶); data reduction: *SAINT* and *XPREP* (Bruker, 2004 ▶); program(s) used to solve structure: *SHELXS97* (Sheldrick, 2008 ▶); program(s) used to refine structure: *SHELXL97* (Sheldrick, 2008 ▶); molecular graphics: *ORTEP-3* (Farrugia (1997 ▶) and *PLATON* (Spek, 2009 ▶); software used to prepare material for publication: *SHELXL97* and *PLATON*.

## Supplementary Material

Crystal structure: contains datablocks global, I. DOI: 10.1107/S160053680905140X/lx2125sup1.cif

Structure factors: contains datablocks I. DOI: 10.1107/S160053680905140X/lx2125Isup2.hkl

Additional supplementary materials:  crystallographic information; 3D view; checkCIF report

## Figures and Tables

**Table 1:** Hydrogen-bond geometry (Å, °)

*D*—H⋯*A*	*D*—H	H⋯*A*	*D*⋯*A*	*D*—H⋯*A*
C4—H4⋯O5	0.93	2.28	2.984 (3)	133
C20—H20⋯O12	0.93	2.24	2.944 (3)	132
N1—H1⋯O8^i^	0.86	2.38	3.126 (2)	146
N2—H2⋯O2^ii^	0.86	2.25	3.088 (2)	163
C21—H21⋯O9^iii^	0.93	2.60	3.431 (3)	149
C23—H23⋯O1^ii^	0.93	2.34	3.254 (3)	166
C29—H29*A*⋯*Cg*1^i^	0.96	2.75	3.530 (3)	139
C31—H31*B*⋯*Cg*2^iv^	0.97	2.69	3.597 (2)	156

Symmetry codes: (i) 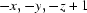; (ii) 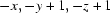; (iii) 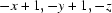; (iv) 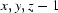. *Cg*1 and *Cg*2 are the centroids of the C3–C8 benzene ring and the N1/C1–C3/C8 pyrrole ring, respectively.

**Fig. 1.**
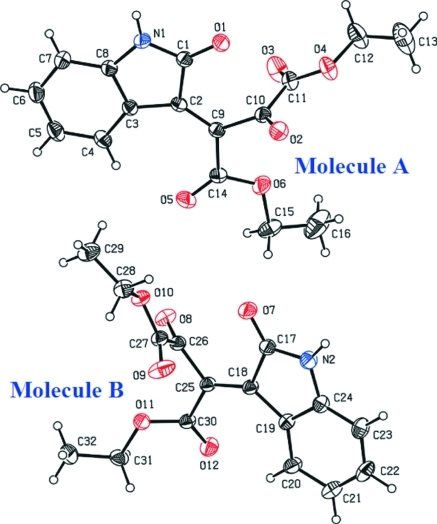
The molecular structure of the title compound with the atom numbering scheme. Displacement ellipsoids are drawn at the 30% probability level. H atoms are presented as a small spheres of arbitrary radius.

**Fig. 2.**
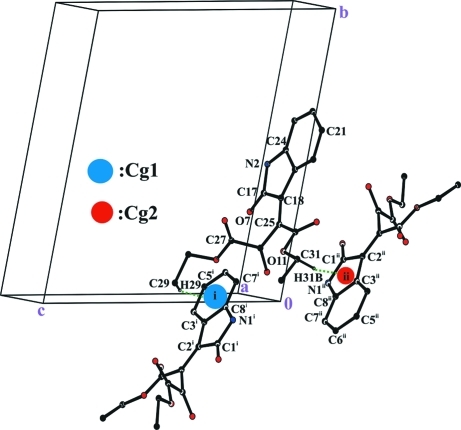
C—H···π interactions (dotted lines) in the structure of the title compound. Cg denotes the ring centroids. [Symmetry code: (i) - x, - y, 1 - z; (ii) x, y, - 1 + z.]

## supplementary crystallographic information

### Comment

Indole derivatives are used as bioactive drugs (Stevenson *et al.*, 2000) and they exhibit anti-allergic, central nervous system depressant and muscle relaxant properties (Harris & Uhle 1960; Ho *et al.*, 1986). Indoles have been proved to display high aldose reductase inhibitory activity (Rajeswaran *et al.*, 1999). Against this background, we report the crystal structure of the title compound, which has two unique molecules in the asymmetric unit (further marked as A & B) (Fig. 1).

In the crystal structure, the bond lengths and angles are found to have normal values (Allen *et al.*,  1987). The indole unit is essentially planar, with a mean deviation of -0.022 (2) Å for A, 0.018 (2) Å for B, respectively, from the least-squares plane defined by the nine constituent atoms. Intramolecular C4—H4···O5 and C20—H20···O12 hydrogen bonds generate S(7) ring motifs (Bernstein *et al.*, 1995). The crystal packing is stabilized by intermolecular C—H···O and N—H···O hydrogen bonds. Atom N1 in the molecule at (*x*, *y*, *z*) donate one proton to atom O8 in the molecule at (-*x*, -*y*, *1 - z*) forming a C(7) chain along *c* axis. Also, atoms N2 and C23 in the molecule at (*x*, *y*, *z*) donate one proton each to atom O2 and O1 in the molecule at (-*x*, *1 - y*, *1 - z*) generating *R*_2_^2^(11) ring motif. The molecules at (*x*, *y*, *z*) and (*1 - x*, *1 - y*, *1 - z*) are linked by C21—H21···O9 hydrogen bonds into cyclic centrosymmetric *R*_2_^2^(18) dimers (Table 1). The molecular packing (Fig. 2) is further stabilized by intermolecular C—H···π interactions; the first between the methyl H atom and the benzene ring of an adjacent molecule, with a C29–H29A···Cg1^i^, the second between the methyl H atom and the pyrrole ring of a neighbouring molecule, with a C31–H31B···Cg2^ii^ (Table 1; Cg1 and Cg2 are the centroids of the C3–C8 benzene ring and the N1/C1/C2/C3/C8 pyrrole ring, respectively).

### Experimental

To a magnetically stirred solution of isatin (2 mmol) and aniline (2 mmol) in ethanol (3 ml) was added drop wise, dimethyl acetylenedicarboxylate (2 mmol, 0.284 g) at room temperature over 10 min. After complete the reaction as indicated by TLC water was added. The reaction mixture was extracted with ethyl acetate (2x30 ml) and the organic layer was separated carefully from the aqueous layer. The combined organic layers were dried over anhydrous Na_2_SO_4_ by which the water present after the extraction can be removed and further, the organic layer is concentrated in vacuum. The crude was purified by column chromatography on silica gel (Merck, 100–200 mesh, ethyl acetate-petroleum ether (15:85). Single crystals suitable for X-ray diffraction were obtained by slow evaporation of a solution of the title compound in ethanol:petroleum ether (3:1)at room temperature.

### Refinement

All H atoms were fixed geometrically and allowed to ride on their parent C atoms, with C—H distances fixed in the range 0.93–0.97 Å, and with *U*_iso_(H) = 1.5*U*_eq_(C) for methyl H 1.2*U*_eq_(C) for other H atoms.

### Crystal data

**Table:**

C_16_H_15_NO_6_	*Z* = 4
*M**_r_* = 317.29	*F*(000) = 664
Triclinic, *P*1	*D*_x_ = 1.365 Mg m^−^^3^
Hall symbol: -P 1	Mo *K*α radiation, λ = 0.71073 Å
*a* = 8.8277 (2) Å	Cell parameters from 8406 reflections
*b* = 13.5365 (4) Å	θ = 1.5–29.6°
*c* = 13.6300 (3) Å	µ = 0.11 mm^−^^1^
α = 96.516 (3)°	*T* = 293 K
β = 102.218 (2)°	Block, colourless
γ = 100.668 (1)°	0.24 × 0.22 × 0.16 mm
*V* = 1544.44 (7) Å^3^	

### Data collection

**Table:**

Bruker APEXII CCD diffractometer	8406 independent reflections
Radiation source: fine-focus sealed tube	5743 reflections with *I* > 2σ(*I*)
graphite	*R*_int_ = 0.028
Detector resolution: 10.0 pixels mm^-1^	θ_max_ = 29.6°, θ_min_ = 1.6°
ω scans	*h* = −12→12
Absorption correction: multi-scan (*SADABS*; Sheldrick, 2001)	*k* = −18→18
*T*_min_ = 0.975, *T*_max_ = 0.983	*l* = −18→18
37712 measured reflections	

### Refinement

**Table:**

Refinement on *F*^2^	Secondary atom site location: difference Fourier map
Least-squares matrix: full	Hydrogen site location: difference Fourier map
*R*[*F*^2^ > 2σ(*F*^2^)] = 0.056	H-atom parameters constrained
*wR*(*F*^2^) = 0.190	*w* = 1/[σ^2^(*F*_o_^2^) + (0.1072*P*)^2^ + 0.3361*P*] where *P* = (*F*_o_^2^ + 2*F*_c_^2^)/3
*S* = 1.02	(Δ/σ)_max_ = 0.001
8406 reflections	Δρ_max_ = 0.60 e Å^−^^3^
420 parameters	Δρ_min_ = −0.35 e Å^−^^3^
0 restraints	Extinction correction: *SHELXL*, Fc^*^=kFc[1+0.001xFc^2^λ^3^/sin(2θ)]^-1/4^
Primary atom site location: structure-invariant direct methods	Extinction coefficient: 0.010 (2)

### Special details

**Table:**

Geometry. All e.s.d.'s (except the e.s.d. in the dihedral angle between two l.s. planes) are estimated using the full covariance matrix. The cell e.s.d.'s are taken into account individually in the estimation of e.s.d.'s in distances, angles and torsion angles; correlations between e.s.d.'s in cell parameters are only used when they are defined by crystal symmetry. An approximate (isotropic) treatment of cell e.s.d.'s is used for estimating e.s.d.'s involving l.s. planes.
Refinement. Refinement of *F*^2^ against ALL reflections. The weighted *R*-factor *wR* and goodness of fit *S* are based on *F*^2^, conventional *R*-factors *R* are based on *F*, with *F* set to zero for negative *F*^2^. The threshold expression of *F*^2^ > σ(*F*^2^) is used only for calculating *R*-factors(gt) *etc*. and is not relevant to the choice of reflections for refinement. *R*-factors based on *F*^2^ are statistically about twice as large as those based on *F*, and *R*- factors based on ALL data will be even larger.

### Fractional atomic coordinates and isotropic or equivalent isotropic displacement parameters (Å^2^)

**Table:**

	*x*	*y*	*z*	*U*_iso_*/*U*_eq_	
C1	−0.0557 (2)	0.14385 (14)	0.77827 (13)	0.0384 (4)	
C2	0.01462 (19)	0.15226 (13)	0.68732 (12)	0.0340 (4)	
C3	0.0893 (2)	0.06556 (13)	0.67808 (12)	0.0354 (4)	
C4	0.1693 (2)	0.02801 (16)	0.61002 (15)	0.0459 (4)	
H4	0.1842	0.0607	0.5553	0.055*	
C5	0.2264 (3)	−0.05904 (17)	0.62498 (18)	0.0553 (5)	
H5	0.2800	−0.0851	0.5797	0.066*	
C6	0.2050 (3)	−0.10727 (17)	0.7058 (2)	0.0584 (6)	
H6	0.2464	−0.1649	0.7149	0.070*	
C7	0.1242 (3)	−0.07275 (16)	0.77380 (18)	0.0521 (5)	
H7	0.1083	−0.1066	0.8277	0.063*	
C8	0.0676 (2)	0.01387 (14)	0.75889 (14)	0.0393 (4)	
C9	−0.0045 (2)	0.23086 (14)	0.63759 (12)	0.0358 (4)	
C10	−0.0871 (2)	0.31007 (14)	0.67537 (13)	0.0375 (4)	
C11	−0.2680 (2)	0.28477 (15)	0.64117 (15)	0.0449 (4)	
C12	−0.4998 (3)	0.3423 (2)	0.6565 (3)	0.0824 (9)	
H12A	−0.5422	0.3143	0.5851	0.099*	
H12B	−0.5456	0.2953	0.6965	0.099*	
C13	−0.5392 (4)	0.4401 (3)	0.6780 (4)	0.1182 (14)	
H13A	−0.4924	0.4688	0.7480	0.177*	
H13B	−0.6524	0.4317	0.6651	0.177*	
H13C	−0.4989	0.4848	0.6352	0.177*	
C14	0.0563 (2)	0.25214 (15)	0.54688 (13)	0.0406 (4)	
C15	0.0267 (3)	0.3480 (2)	0.41055 (17)	0.0647 (6)	
H15A	0.0326	0.2891	0.3651	0.078*	
H15B	0.1287	0.3955	0.4280	0.078*	
C16	−0.1013 (5)	0.3972 (3)	0.3625 (3)	0.1078 (12)	
H16A	−0.2008	0.3487	0.3445	0.162*	
H16B	−0.0779	0.4208	0.3024	0.162*	
H16C	−0.1077	0.4539	0.4095	0.162*	
C17	0.1535 (2)	0.36702 (14)	0.12817 (13)	0.0400 (4)	
C18	0.2254 (2)	0.34775 (13)	0.03846 (12)	0.0347 (4)	
C19	0.2462 (2)	0.44351 (13)	−0.00188 (12)	0.0367 (4)	
C20	0.3048 (3)	0.47612 (15)	−0.08198 (15)	0.0464 (4)	
H20	0.3385	0.4314	−0.1254	0.056*	
C21	0.3125 (3)	0.57598 (16)	−0.09617 (16)	0.0538 (5)	
H21	0.3528	0.5989	−0.1492	0.065*	
C22	0.2612 (3)	0.64208 (15)	−0.03279 (17)	0.0551 (5)	
H22	0.2664	0.7089	−0.0445	0.066*	
C23	0.2026 (3)	0.61209 (15)	0.04728 (16)	0.0518 (5)	
H23	0.1682	0.6572	0.0900	0.062*	
C24	0.1967 (2)	0.51261 (14)	0.06180 (13)	0.0397 (4)	
C25	0.2607 (2)	0.25640 (13)	0.01784 (12)	0.0340 (4)	
C26	0.2336 (2)	0.17683 (13)	0.08415 (12)	0.0354 (4)	
C27	0.3438 (2)	0.19955 (14)	0.19117 (13)	0.0389 (4)	
C28	0.3865 (3)	0.15228 (16)	0.35426 (13)	0.0483 (5)	
H28A	0.3549	0.2050	0.3947	0.058*	
H28B	0.4988	0.1736	0.3574	0.058*	
C29	0.3561 (3)	0.05597 (18)	0.39389 (15)	0.0580 (6)	
H29A	0.2444	0.0343	0.3885	0.087*	
H29B	0.4123	0.0656	0.4639	0.087*	
H29C	0.3919	0.0050	0.3552	0.087*	
C30	0.3304 (2)	0.22821 (13)	−0.06912 (12)	0.0350 (4)	
C31	0.4944 (2)	0.12998 (16)	−0.12933 (15)	0.0455 (4)	
H31A	0.5632	0.1894	−0.1420	0.055*	
H31B	0.4154	0.1003	−0.1918	0.055*	
C32	0.5884 (3)	0.0553 (2)	−0.0959 (2)	0.0707 (7)	
H32A	0.6597	0.0830	−0.0310	0.106*	
H32B	0.6483	0.0402	−0.1447	0.106*	
H32C	0.5185	−0.0061	−0.0902	0.106*	
N1	−0.01684 (19)	0.06241 (12)	0.81703 (12)	0.0428 (4)	
H1	−0.0411	0.0429	0.8707	0.051*	
N2	0.1433 (2)	0.46565 (12)	0.13772 (12)	0.0455 (4)	
H2	0.1083	0.4957	0.1846	0.055*	
O1	−0.13388 (18)	0.20008 (11)	0.80943 (11)	0.0527 (4)	
O2	−0.01530 (17)	0.39110 (10)	0.72271 (11)	0.0502 (3)	
O3	−0.34030 (19)	0.21042 (13)	0.58261 (14)	0.0718 (5)	
O4	−0.32745 (17)	0.35627 (12)	0.68209 (12)	0.0605 (4)	
O5	0.15638 (19)	0.21704 (14)	0.51818 (12)	0.0631 (4)	
O6	−0.0174 (2)	0.31791 (12)	0.50174 (11)	0.0555 (4)	
O7	0.1126 (2)	0.30431 (12)	0.18008 (11)	0.0563 (4)	
O8	0.14135 (17)	0.09712 (10)	0.05381 (10)	0.0483 (3)	
O9	0.4607 (2)	0.26501 (14)	0.21396 (12)	0.0705 (5)	
O10	0.29497 (15)	0.13619 (10)	0.24918 (9)	0.0413 (3)	
O11	0.41725 (16)	0.15912 (10)	−0.04989 (9)	0.0423 (3)	
O12	0.30961 (18)	0.26196 (11)	−0.14685 (9)	0.0496 (4)	

### Atomic displacement parameters (Å^2^)

**Table:**

	*U*^11^	*U*^22^	*U*^33^	*U*^12^	*U*^13^	*U*^23^
C1	0.0429 (9)	0.0390 (9)	0.0369 (8)	0.0105 (8)	0.0155 (7)	0.0065 (7)
C2	0.0335 (8)	0.0380 (9)	0.0318 (7)	0.0074 (7)	0.0114 (6)	0.0038 (6)
C3	0.0346 (8)	0.0354 (9)	0.0361 (8)	0.0080 (7)	0.0094 (7)	0.0032 (7)
C4	0.0456 (10)	0.0513 (12)	0.0450 (10)	0.0155 (9)	0.0174 (8)	0.0034 (8)
C5	0.0517 (11)	0.0540 (13)	0.0649 (13)	0.0211 (10)	0.0220 (10)	−0.0025 (10)
C6	0.0535 (12)	0.0420 (11)	0.0850 (16)	0.0197 (10)	0.0209 (11)	0.0082 (11)
C7	0.0523 (11)	0.0416 (11)	0.0698 (13)	0.0154 (9)	0.0202 (10)	0.0193 (10)
C8	0.0357 (8)	0.0377 (9)	0.0454 (9)	0.0073 (7)	0.0118 (7)	0.0068 (7)
C9	0.0366 (8)	0.0397 (9)	0.0324 (7)	0.0086 (7)	0.0113 (6)	0.0048 (7)
C10	0.0432 (9)	0.0374 (9)	0.0350 (8)	0.0104 (7)	0.0133 (7)	0.0085 (7)
C11	0.0441 (10)	0.0450 (11)	0.0460 (9)	0.0147 (8)	0.0095 (8)	0.0029 (8)
C12	0.0502 (13)	0.093 (2)	0.106 (2)	0.0313 (14)	0.0160 (14)	0.0034 (17)
C13	0.078 (2)	0.118 (3)	0.166 (4)	0.054 (2)	0.027 (2)	0.001 (3)
C14	0.0425 (9)	0.0434 (10)	0.0378 (8)	0.0080 (8)	0.0138 (7)	0.0091 (7)
C15	0.0844 (17)	0.0679 (15)	0.0454 (11)	0.0082 (13)	0.0214 (11)	0.0256 (10)
C16	0.163 (4)	0.086 (2)	0.0790 (19)	0.032 (2)	0.020 (2)	0.0371 (17)
C17	0.0483 (10)	0.0405 (10)	0.0373 (8)	0.0134 (8)	0.0189 (7)	0.0083 (7)
C18	0.0416 (9)	0.0328 (9)	0.0319 (7)	0.0073 (7)	0.0133 (7)	0.0070 (6)
C19	0.0415 (9)	0.0319 (9)	0.0357 (8)	0.0057 (7)	0.0082 (7)	0.0063 (7)
C20	0.0582 (11)	0.0401 (10)	0.0445 (9)	0.0077 (9)	0.0197 (9)	0.0125 (8)
C21	0.0637 (13)	0.0439 (11)	0.0523 (11)	0.0011 (10)	0.0137 (10)	0.0193 (9)
C22	0.0655 (13)	0.0311 (10)	0.0617 (12)	0.0019 (9)	0.0044 (10)	0.0128 (9)
C23	0.0633 (12)	0.0327 (10)	0.0551 (11)	0.0096 (9)	0.0089 (10)	0.0010 (8)
C24	0.0445 (9)	0.0349 (9)	0.0372 (8)	0.0072 (7)	0.0068 (7)	0.0029 (7)
C25	0.0392 (8)	0.0329 (9)	0.0316 (7)	0.0062 (7)	0.0130 (6)	0.0066 (6)
C26	0.0422 (9)	0.0335 (9)	0.0344 (8)	0.0084 (7)	0.0168 (7)	0.0070 (6)
C27	0.0434 (9)	0.0396 (10)	0.0368 (8)	0.0089 (8)	0.0138 (7)	0.0112 (7)
C28	0.0538 (11)	0.0560 (12)	0.0330 (8)	0.0094 (9)	0.0070 (8)	0.0093 (8)
C29	0.0618 (13)	0.0688 (15)	0.0410 (10)	0.0053 (11)	0.0067 (9)	0.0233 (10)
C30	0.0429 (9)	0.0309 (8)	0.0323 (7)	0.0051 (7)	0.0137 (7)	0.0050 (6)
C31	0.0510 (10)	0.0444 (11)	0.0460 (10)	0.0102 (9)	0.0249 (8)	0.0022 (8)
C32	0.0883 (18)	0.0684 (16)	0.0830 (17)	0.0424 (14)	0.0524 (15)	0.0236 (13)
N1	0.0522 (9)	0.0441 (9)	0.0410 (8)	0.0150 (7)	0.0219 (7)	0.0155 (7)
N2	0.0618 (10)	0.0400 (9)	0.0407 (8)	0.0167 (8)	0.0223 (7)	0.0029 (6)
O1	0.0691 (9)	0.0518 (9)	0.0550 (8)	0.0278 (7)	0.0369 (7)	0.0154 (7)
O2	0.0514 (8)	0.0391 (8)	0.0563 (8)	0.0049 (6)	0.0133 (6)	−0.0015 (6)
O3	0.0503 (8)	0.0646 (11)	0.0850 (12)	0.0119 (8)	0.0004 (8)	−0.0223 (9)
O4	0.0476 (8)	0.0597 (10)	0.0724 (10)	0.0225 (7)	0.0114 (7)	−0.0093 (8)
O5	0.0635 (9)	0.0827 (12)	0.0651 (9)	0.0311 (9)	0.0400 (8)	0.0308 (9)
O6	0.0756 (10)	0.0593 (9)	0.0458 (7)	0.0270 (8)	0.0261 (7)	0.0246 (7)
O7	0.0802 (10)	0.0532 (9)	0.0542 (8)	0.0248 (8)	0.0408 (8)	0.0211 (7)
O8	0.0620 (8)	0.0392 (7)	0.0399 (6)	−0.0014 (6)	0.0129 (6)	0.0089 (5)
O9	0.0624 (9)	0.0742 (11)	0.0580 (9)	−0.0204 (8)	−0.0011 (7)	0.0279 (8)
O10	0.0473 (7)	0.0430 (7)	0.0335 (6)	0.0044 (6)	0.0100 (5)	0.0130 (5)
O11	0.0536 (8)	0.0422 (7)	0.0402 (6)	0.0180 (6)	0.0231 (6)	0.0094 (5)
O12	0.0725 (9)	0.0501 (8)	0.0347 (6)	0.0214 (7)	0.0210 (6)	0.0120 (6)

### Geometric parameters (Å, °)

**Table:**

C1—O1	1.217 (2)	C17—N2	1.348 (2)
C1—N1	1.344 (2)	C17—C18	1.511 (2)
C1—C2	1.505 (2)	C18—C25	1.344 (2)
C2—C9	1.342 (2)	C18—C19	1.460 (2)
C2—C3	1.455 (2)	C19—C20	1.385 (3)
C3—C4	1.386 (3)	C19—C24	1.396 (3)
C3—C8	1.398 (3)	C20—C21	1.379 (3)
C4—C5	1.384 (3)	C20—H20	0.9300
C4—H4	0.9300	C21—C22	1.375 (3)
C5—C6	1.372 (3)	C21—H21	0.9300
C5—H5	0.9300	C22—C23	1.373 (3)
C6—C7	1.376 (3)	C22—H22	0.9300
C6—H6	0.9300	C23—C24	1.376 (3)
C7—C8	1.376 (3)	C23—H23	0.9300
C7—H7	0.9300	C24—N2	1.394 (2)
C8—N1	1.389 (2)	C25—C30	1.490 (2)
C9—C14	1.485 (2)	C25—C26	1.500 (2)
C9—C10	1.506 (3)	C26—O8	1.196 (2)
C10—O2	1.196 (2)	C26—C27	1.535 (2)
C10—C11	1.526 (3)	C27—O9	1.187 (2)
C11—O3	1.192 (2)	C27—O10	1.307 (2)
C11—O4	1.309 (2)	C28—O10	1.458 (2)
C12—C13	1.443 (4)	C28—C29	1.468 (3)
C12—O4	1.458 (3)	C28—H28A	0.9700
C12—H12A	0.9700	C28—H28B	0.9700
C12—H12B	0.9700	C29—H29A	0.9600
C13—H13A	0.9600	C29—H29B	0.9600
C13—H13B	0.9600	C29—H29C	0.9600
C13—H13C	0.9600	C30—O12	1.193 (2)
C14—O5	1.191 (2)	C30—O11	1.330 (2)
C14—O6	1.328 (2)	C31—O11	1.452 (2)
C15—O6	1.457 (2)	C31—C32	1.475 (3)
C15—C16	1.487 (4)	C31—H31A	0.9700
C15—H15A	0.9700	C31—H31B	0.9700
C15—H15B	0.9700	C32—H32A	0.9600
C16—H16A	0.9600	C32—H32B	0.9600
C16—H16B	0.9600	C32—H32C	0.9600
C16—H16C	0.9600	N1—H1	0.8600
C17—O7	1.217 (2)	N2—H2	0.8600
			
O1—C1—N1	127.09 (17)	C19—C18—C17	105.58 (15)
O1—C1—C2	125.97 (17)	C20—C19—C24	119.05 (17)
N1—C1—C2	106.93 (15)	C20—C19—C18	134.57 (17)
C9—C2—C3	136.93 (16)	C24—C19—C18	106.35 (15)
C9—C2—C1	117.74 (16)	C21—C20—C19	118.9 (2)
C3—C2—C1	105.33 (14)	C21—C20—H20	120.6
C4—C3—C8	119.03 (17)	C19—C20—H20	120.6
C4—C3—C2	134.46 (17)	C22—C21—C20	120.8 (2)
C8—C3—C2	106.51 (15)	C22—C21—H21	119.6
C5—C4—C3	118.80 (19)	C20—C21—H21	119.6
C5—C4—H4	120.6	C23—C22—C21	121.74 (19)
C3—C4—H4	120.6	C23—C22—H22	119.1
C6—C5—C4	120.8 (2)	C21—C22—H22	119.1
C6—C5—H5	119.6	C22—C23—C24	117.3 (2)
C4—C5—H5	119.6	C22—C23—H23	121.4
C5—C6—C7	121.8 (2)	C24—C23—H23	121.4
C5—C6—H6	119.1	C23—C24—N2	127.40 (18)
C7—C6—H6	119.1	C23—C24—C19	122.28 (18)
C6—C7—C8	117.3 (2)	N2—C24—C19	110.32 (16)
C6—C7—H7	121.3	C18—C25—C30	123.16 (15)
C8—C7—H7	121.3	C18—C25—C26	120.70 (15)
C7—C8—N1	127.58 (18)	C30—C25—C26	116.13 (15)
C7—C8—C3	122.25 (18)	O8—C26—C25	122.65 (15)
N1—C8—C3	110.17 (15)	O8—C26—C27	121.44 (15)
C2—C9—C14	125.62 (17)	C25—C26—C27	115.65 (14)
C2—C9—C10	120.51 (15)	O9—C27—O10	126.28 (17)
C14—C9—C10	113.80 (15)	O9—C27—C26	122.21 (16)
O2—C10—C9	122.08 (16)	O10—C27—C26	111.46 (15)
O2—C10—C11	121.74 (17)	O10—C28—C29	108.15 (16)
C9—C10—C11	115.94 (15)	O10—C28—H28A	110.1
O3—C11—O4	126.54 (19)	C29—C28—H28A	110.1
O3—C11—C10	122.63 (18)	O10—C28—H28B	110.1
O4—C11—C10	110.80 (16)	C29—C28—H28B	110.1
C13—C12—O4	108.4 (3)	H28A—C28—H28B	108.4
C13—C12—H12A	110.0	C28—C29—H29A	109.5
O4—C12—H12A	110.0	C28—C29—H29B	109.5
C13—C12—H12B	110.0	H29A—C29—H29B	109.5
O4—C12—H12B	110.0	C28—C29—H29C	109.5
H12A—C12—H12B	108.4	H29A—C29—H29C	109.5
C12—C13—H13A	109.5	H29B—C29—H29C	109.5
C12—C13—H13B	109.5	O12—C30—O11	123.95 (16)
H13A—C13—H13B	109.5	O12—C30—C25	124.67 (17)
C12—C13—H13C	109.5	O11—C30—C25	111.38 (14)
H13A—C13—H13C	109.5	O11—C31—C32	108.50 (17)
H13B—C13—H13C	109.5	O11—C31—H31A	110.0
O5—C14—O6	124.35 (18)	C32—C31—H31A	110.0
O5—C14—C9	126.15 (18)	O11—C31—H31B	110.0
O6—C14—C9	109.50 (16)	C32—C31—H31B	110.0
O6—C15—C16	105.1 (2)	H31A—C31—H31B	108.4
O6—C15—H15A	110.7	C31—C32—H32A	109.5
C16—C15—H15A	110.7	C31—C32—H32B	109.5
O6—C15—H15B	110.7	H32A—C32—H32B	109.5
C16—C15—H15B	110.7	C31—C32—H32C	109.5
H15A—C15—H15B	108.8	H32A—C32—H32C	109.5
C15—C16—H16A	109.5	H32B—C32—H32C	109.5
C15—C16—H16B	109.5	C1—N1—C8	111.02 (15)
H16A—C16—H16B	109.5	C1—N1—H1	124.5
C15—C16—H16C	109.5	C8—N1—H1	124.5
H16A—C16—H16C	109.5	C17—N2—C24	111.23 (15)
H16B—C16—H16C	109.5	C17—N2—H2	124.4
O7—C17—N2	127.76 (17)	C24—N2—H2	124.4
O7—C17—C18	125.78 (17)	C11—O4—C12	117.01 (18)
N2—C17—C18	106.46 (15)	C14—O6—C15	117.48 (17)
C25—C18—C19	136.02 (16)	C27—O10—C28	116.77 (14)
C25—C18—C17	118.30 (15)	C30—O11—C31	115.03 (14)
			
O1—C1—C2—C9	2.7 (3)	C19—C20—C21—C22	−0.7 (3)
N1—C1—C2—C9	−177.88 (15)	C20—C21—C22—C23	0.8 (3)
O1—C1—C2—C3	−177.12 (18)	C21—C22—C23—C24	−0.1 (3)
N1—C1—C2—C3	2.26 (19)	C22—C23—C24—N2	179.56 (19)
C9—C2—C3—C4	−2.0 (4)	C22—C23—C24—C19	−0.6 (3)
C1—C2—C3—C4	177.81 (19)	C20—C19—C24—C23	0.6 (3)
C9—C2—C3—C8	178.5 (2)	C18—C19—C24—C23	178.86 (18)
C1—C2—C3—C8	−1.66 (18)	C20—C19—C24—N2	−179.51 (16)
C8—C3—C4—C5	−0.6 (3)	C18—C19—C24—N2	−1.27 (19)
C2—C3—C4—C5	179.95 (19)	C19—C18—C25—C30	−4.9 (3)
C3—C4—C5—C6	−0.2 (3)	C17—C18—C25—C30	179.33 (15)
C4—C5—C6—C7	1.2 (3)	C19—C18—C25—C26	174.04 (18)
C5—C6—C7—C8	−1.3 (3)	C17—C18—C25—C26	−1.7 (2)
C6—C7—C8—N1	179.91 (19)	C18—C25—C26—O8	116.3 (2)
C6—C7—C8—C3	0.4 (3)	C30—C25—C26—O8	−64.7 (2)
C4—C3—C8—C7	0.6 (3)	C18—C25—C26—C27	−69.4 (2)
C2—C3—C8—C7	−179.88 (17)	C30—C25—C26—C27	109.57 (17)
C4—C3—C8—N1	−179.04 (16)	O8—C26—C27—O9	160.7 (2)
C2—C3—C8—N1	0.53 (19)	C25—C26—C27—O9	−13.6 (3)
C3—C2—C9—C14	−0.4 (3)	O8—C26—C27—O10	−16.8 (2)
C1—C2—C9—C14	179.78 (15)	C25—C26—C27—O10	168.85 (15)
C3—C2—C9—C10	−177.37 (18)	C18—C25—C30—O12	−28.9 (3)
C1—C2—C9—C10	2.8 (2)	C26—C25—C30—O12	152.14 (18)
C2—C9—C10—O2	100.8 (2)	C18—C25—C30—O11	152.05 (16)
C14—C9—C10—O2	−76.5 (2)	C26—C25—C30—O11	−26.9 (2)
C2—C9—C10—C11	−84.7 (2)	O1—C1—N1—C8	177.35 (19)
C14—C9—C10—C11	97.97 (18)	C2—C1—N1—C8	−2.0 (2)
O2—C10—C11—O3	169.5 (2)	C7—C8—N1—C1	−178.57 (19)
C9—C10—C11—O3	−5.0 (3)	C3—C8—N1—C1	1.0 (2)
O2—C10—C11—O4	−8.5 (3)	O7—C17—N2—C24	−177.8 (2)
C9—C10—C11—O4	177.00 (16)	C18—C17—N2—C24	1.8 (2)
C2—C9—C14—O5	−16.8 (3)	C23—C24—N2—C17	179.5 (2)
C10—C9—C14—O5	160.4 (2)	C19—C24—N2—C17	−0.4 (2)
C2—C9—C14—O6	163.70 (17)	O3—C11—O4—C12	2.3 (4)
C10—C9—C14—O6	−19.2 (2)	C10—C11—O4—C12	−179.8 (2)
O7—C17—C18—C25	−5.9 (3)	C13—C12—O4—C11	−160.4 (3)
N2—C17—C18—C25	174.52 (16)	O5—C14—O6—C15	0.6 (3)
O7—C17—C18—C19	177.09 (19)	C9—C14—O6—C15	−179.88 (17)
N2—C17—C18—C19	−2.45 (19)	C16—C15—O6—C14	165.1 (2)
C25—C18—C19—C20	3.9 (4)	O9—C27—O10—C28	4.9 (3)
C17—C18—C19—C20	−179.9 (2)	C26—C27—O10—C28	−177.72 (15)
C25—C18—C19—C24	−173.9 (2)	C29—C28—O10—C27	−157.96 (18)
C17—C18—C19—C24	2.22 (18)	O12—C30—O11—C31	3.1 (3)
C24—C19—C20—C21	0.1 (3)	C25—C30—O11—C31	−177.85 (14)
C18—C19—C20—C21	−177.6 (2)	C32—C31—O11—C30	178.71 (18)

### Hydrogen-bond geometry (Å, °)

**Table:**

*D*—H···*A*	*D*—H	H···*A*	*D*···*A*	*D*—H···*A*
C4—H4···O5	0.93	2.28	2.984 (3)	133
C20—H20···O12	0.93	2.24	2.944 (3)	132
N1—H1···O8^i^	0.86	2.38	3.126 (2)	146
N2—H2···O2^ii^	0.86	2.25	3.088 (2)	163
C21—H21···O9^iii^	0.93	2.60	3.431 (3)	149
C23—H23···O1^ii^	0.93	2.34	3.254 (3)	166
C29—H29A···Cg1^i^	0.96	2.75	3.530 (3)	139
C31—H31B···Cg2^iv^	0.97	2.69	3.597 (2)	156

Symmetry codes:  (i) −*x*, −*y*, −*z*+1; (ii) −*x*, −*y*+1, −*z*+1; (iii) −*x*+1, −*y*+1, −*z*; (iv) *x*, *y*, *z*−1.
